# Sagittal Alignment Correction and the Extent of Intervertebral Distraction as Factors Associated with Postoperative Radiculitis Following Anterior Lumbar Interbody Fusion

**DOI:** 10.3390/jcm15124707

**Published:** 2026-06-17

**Authors:** Paula Lavezzolo, Francesco Caiazzo, Lucas Capo, Daniel Alveal-Mellado, Judith Salat-Batlle, Andreas Leidinger, Juan Bago

**Affiliations:** 1 Instituto Quirúrgico Spanò, Sagrada Familia Medical Center, 08022 Barcelona, Spain; plavezzolo@iqspano.com (P.L.);; 2 Fundación Privada Spanò, 08022 Barcelona, Spain

**Keywords:** retrospective study, global alignment and proportion scoring, lumbar fusion, complications, anterior lumbar interbody fusion

## Abstract

**Background/Objectives:** To investigate the prevalence and associated factors of postoperative radiculitis in patients treated with Anterior Lumbar Interbody Fusion (ALIF) in L4–L5 or L5–S1 utilizing lumbopelvic sagittal parameters and ideal alignment values calculated with GAP score components. **Methods:** A retrospective review using Natural Language Processing (NLP) for automated data extraction from clinical notes was conducted. 61 adult patients were included in the analysis. Postoperative radiculitis was defined as the new onset of unilateral or bilateral pain in the L4–S1 radicular territory with preserved motor function. Clinical parameters (Oswestry Disability Index), radiographic parameters (lumbopelvic sagittal alignment using GAP score components), and surgical factors (cage size and disc height modification) were evaluated and subsequently entered into a multivariable logistic regression analysis. **Results:** Postoperative radiculitis occurred in 29.5% of patients, with symptoms primarily manifesting within the first six weeks following surgery and lasting up to six months. Patients in the Pain group showed notable differences in pre- and postoperative GAP score parameters, specifically higher values for relative lumbar lordosis (RLL) and relative pelvic version (RPV). Furthermore, multivariable logistic regression identified postoperative RLL and the anterior disc height (ADH) ratio to be independently associated with the development of radiculitis. **Conclusions:** Excessive disc space enlargement during ALIF is associated with a higher likelihood of postoperative radiculitis, particularly in patients with pre-existing near-ideal lumbar alignment. To mitigate this iatrogenic complication in this group, the degree of correction must be individualized. This tailored approach should incorporate parameters from the GAP score and a careful assessment of the relationship between the anterior disc height and the vertebral body.

## 1. Introduction

Anterior lumbar interbody fusion (ALIF) is an increasingly common lumbar fusion approach. This procedure requires careful intraoperative navigation of retroperitoneal structures, including the sympathetic chain, genitourinary nerves, and the lumbosacral plexus [1]. The approach carries a risk of neurological complications, including nerve root injury, which presents as radiculopathy or neurological deficit. Reported incidence rates for these complications vary considerably, ranging from 0.1% to 38.4% [2].

Postoperative pain, manifesting as lower limb neuralgic symptoms, develops in a significant number of patients days to weeks after surgery [3]. This pain often occurs without detectable structural nerve damage, complicating patient recovery [4]. The absence of a clear structural etiology and the wide terminological variation complicate the comprehensive analysis of this entity. To address this, Goldman et al. [5] recently proposed the term “postoperative radiculitis”, which they suggest is an adequate descriptor for transient radicular pain that lacks structural pathology or motor weakness and typically emerges after the patient’s preoperative symptoms have resolved.

Postoperative radiculitis has been associated with several surgical factors, including mechanical stretching of the nerve root during the ALIF procedure, increased disc height due to implant insertion, and the subsequent correction of segmental lordosis at the operated level [4,6,7]. However, a clear identification of other potential associated factors, such as pre-existing patient characteristics or the achieved degree of lumbopelvic correction, remains elusive.

Current surgical practice favors the use of larger or hyperlordotic cages to achieve lumbopelvic lordosis correction, an approach supported by radiographic and clinical outcomes [8,9]. However, caution is warranted, since the insertion of excessively lordotic cages may not enhance the restoration of lumbar lordosis but increase the risk of cage subsidence and neurological complications [10].

The Global Alignment and Proportion (GAP) score was designed to predict postoperative mechanical complications in adult spine deformity surgery and a separate analysis of its components is helpful for studying a patient’s sagittal alignment [11,12]. For lumbar spine fusion, analyzing factors related to lumbopelvic alignment is particularly relevant, including relative pelvic version (RPV), relative lumbar lordosis (RLL), and lordosis distribution index (LDI). The methodology compares the patient’s profile to an ideal alignment individualized by their invariant pelvic incidence (PI) [13]. Additionally, it strongly correlates with health-related quality of life measurements [14].

While effective for alignment assessment, the relationship between these GAP score components and its utility to estimate postoperative complications following ALIF has yet to be investigated.

The objectives of this study are: (1) to determine the incidence of postoperative radiculitis in patients treated with lumbosacral ALIF (with or without posterior fixation); (2) to compare patients with postoperative radiculitis (Pain group) with those who did not report pain (Non-Pain group) with respect to actual lumbopelvic sagittal parameters and ideal alignment values calculated with GAP score components; and (3) to analyze the variables associated with the occurrence of postoperative radiculitis.

## 2. Materials and Methods

### 2.1. Study Design and Participants

A retrospective review of our center’s clinical records was conducted. Surgery reports from January 2021 to July 2024 were reviewed. Inclusion criteria considered adult patients (>18 years old) with a diagnosis of degenerative disc disease and/or spondylolisthesis who subsequently underwent single-segment ALIF either at the L4–L5 or L5–S1 level. A minimum follow-up period of 12 months, both clinically and radiographically, was required.

Exclusion criteria included prior surgery at the index level or incomplete medical records. Additionally, patients were excluded if lower limb pain was accompanied by an objectively demonstrated neurological deficit.

The study protocol was approved by the Institutional Ethics Committee (No. CEIm-51/23), and written informed consent was obtained from all participants.

Postoperative radiculitis was defined as the new onset of unilateral or bilateral pain in the L4–S1 radicular territory with preserved motor function, diagnosed during postoperative follow-up assessments [5].

To maintain rigorous data privacy, clinical note extraction was conducted locally through a Natural Language Processing (NLP) pipeline orchestrated with Python (v 3.14.3). A local Large Language Model (LLM), LLaMA 3.1 8B (Meta Platforms Inc., Menlo Park, CA, USA), was configured with a specific instruction set to analyze unstructured clinical texts for the presence of new-onset postoperative radiculitis. Crucially, the clinical notes contained records from multiple postoperative visits for each patient, enabling the LLM to longitudinally track the precise onset of pain manifestation. This evaluation focused on patient mentions of lower limb pain or burning-like pain in the L4–S1 radicular territory. Extraction was performed using schema-based, few-shot prompting. To ensure the precision of the output, “golden examples” (including descriptors like “distraction-related pain,” “burning-like pain,” and “new-onset lower limb pain in the postoperative period”) were utilized. Every case identified by the LLM subsequently underwent manual review by an experienced researcher. Details on specific prompts are available in Supplementary Materials S1.

All patients underwent surgery by the same surgeon using a similar technique. ALIF was performed with the patient in the supine position. A midline infraumbilical incision and retroperitoneal approach to the anterior lumbar spine was carried out. After complete discectomy, an ALIF cage with pre-planned dimensions and lordotic angle was implanted to restore disc height. The cage was filled with demineralized bone matrix.

For cases requiring supplementary posterior instrumentation, the patient was repositioned to the prone position during the same surgical procedure.

Implant dimensions were determined on an individual basis. Specifically, the disc width at the anterior disc space was measured using magnetic resonance imaging (MRI) at the level of the anterior longitudinal ligament. The lateral portions of the annulus fibrosus were excluded from this measurement to avoid overestimation of the disc width and potential risk of nerve root impingement. Cage angulation was determined using the GAP score methodology [11]. Thus, actual lumbopelvic sagittal alignment was compared with ideal values.

Postoperative analgesia utilized a two-phase multimodal protocol. During the first 48 h, an intravenous multimodal elastomeric pump (ketorolac, tramadol, and metamizole) was administered. After pump removal, patients transitioned to a minimum two-week oral regimen of dexketoprofen, paracetamol and gabapentin. Tramadol was indicated in case of pain exacerbation.

Mobilization was initiated within 24 h post-surgery. Upon discharge, patients were prescribed a lumbar brace for 6 weeks. Postoperative radiological follow-up included a standing full-spine radiograph at 48 h, 6 weeks, 6 months, and 12 months after surgery. Dynamic lumbar radiographs were performed at 3 months to assess implant integrity and detect early hardware failure or instability. At the 1-year follow-up, computed tomography (CT) and MRI scans were performed to evaluate fusion, alignment, and adjacent segment status.

### 2.2. Data Collection and Radiographic Analysis

Radiographic parameters, including PI, pelvic tilt (PT), sacral slope (SS), L1–S1 lumbar lordosis (LL), and L4–S1 lumbar lordosis (L4S1), were measured on full-body sagittal radiographs. The adequacy of lumbopelvic sagittal parameters relative to ideal, PI-adjusted values was determined using the GAP score components. These components included the RPV, calculated as the measured SS minus the ideal SS (aligned −7° to +5°); the RLL, defined as the measured LL minus the ideal LL (aligned −14° to +11°); and the LDI, which is the ratio of L4–S1 lordosis to LL, expressed as a percentage (aligned 50% to 80%).

**Figure 1:**
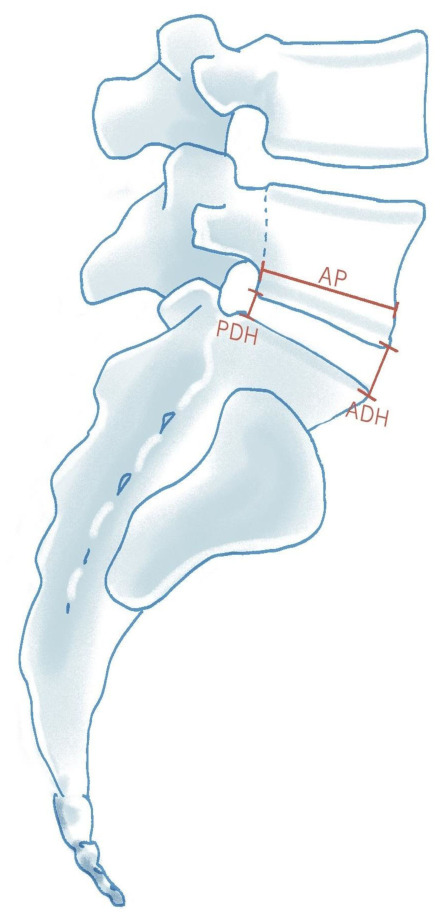
Schematic representation of the ratio calculation for anterior disc height (ADH) and posterior disc height (PDH). AP, anteroposterior length.

To account for variable magnification and inconsistent calibration in outside radiographs, indirect ratio-based measurements were used. Disc heights were normalized against the superior vertebral body’s anteroposterior (AP) length, measured at the vertical midpoint. This yielded the following ratio variables (Figure 1):

(i) Anterior Disc Height (ADH) ratio: The anterior disc height (measured from the anterior corners of L4–L5 or L5–S1 vertebral bodies) divided by the superior vertebral body’s AP length.

(ii) Posterior Disc Height (PDH) ratio: The posterior disc height (measured from the posterior corners of L4–L5 or L5–S1 vertebral bodies) divided by the superior vertebral body’s AP length.

To assess the reliability of the radiological measurements, two independent observers, blinded to clinical outcomes, measured the X-rays at two separate times in a subgroup of 50 patients.

### 2.3. Statistical Analysis

The cohort was summarized using descriptive statistics. Continuous variables are presented as the mean ± standard deviation (SD), and categorical variables as frequencies and percentages. The Shapiro–Wilk test was used to assess data distribution normality. To identify potential factors associated with postoperative radiculitis, the cohort was divided into Pain and Non-Pain groups. Differences were evaluated using Student’s *t*-test or Mann–Whitney U test for continuous variables, and the Chi-square or Fisher’s exact test for categorical variables.

Univariable logistic regression was performed to evaluate the association between individual parameters and postoperative radiculitis. Subsequently, multivariable logistic regression analysis was conducted using the Firth’s penalized likelihood logistic regression to mitigate bias from the small sample and low event count [15]. Candidate models were iteratively constructed using the variables previously selected based on their univariable significance and a priori clinical importance. The final multivariable model was assessed using the variance inflation factor (VIF) and selected using the Akaike Information Criterion (AIC) [16].

All statistical analyses were performed using R software (version 4.3.1; R Foundation for Statistical Computing, Vienna, Austria). A *p*-value < 0.05 was considered statistically significant.

## 3. Results

A total of 127 ALIF procedures were performed during the study period. After excluding 42 cases of multi-segment treatment and 21 revision surgeries, 64 patients who had undergone single-segment ALIF remained eligible. Three patients were subsequently excluded from this cohort: one due to missing data and two who developed a postoperative motor deficit. In one case, an L5 deficit was observed postoperatively. Imaging (CT scan and MRI) showed no structural cause, and the patient had partial improvement within the first year. The second patient’s deficit was caused by a malpositioned pedicle screw; the patient underwent reoperation and subsequently experienced functional improvement. This resulted in a neurological injury rate of 2/64 (3.1%). Consequently, 61 patients (34 females, 27 males) were included in the final analysis.

The cohort’s mean age was 47.5 ± 9.1 years. The L5–S1 segment was the most common level treated, and supplemental posterior fixation was utilized in 65.6% of cases (Table 1).

**Figure 2:**
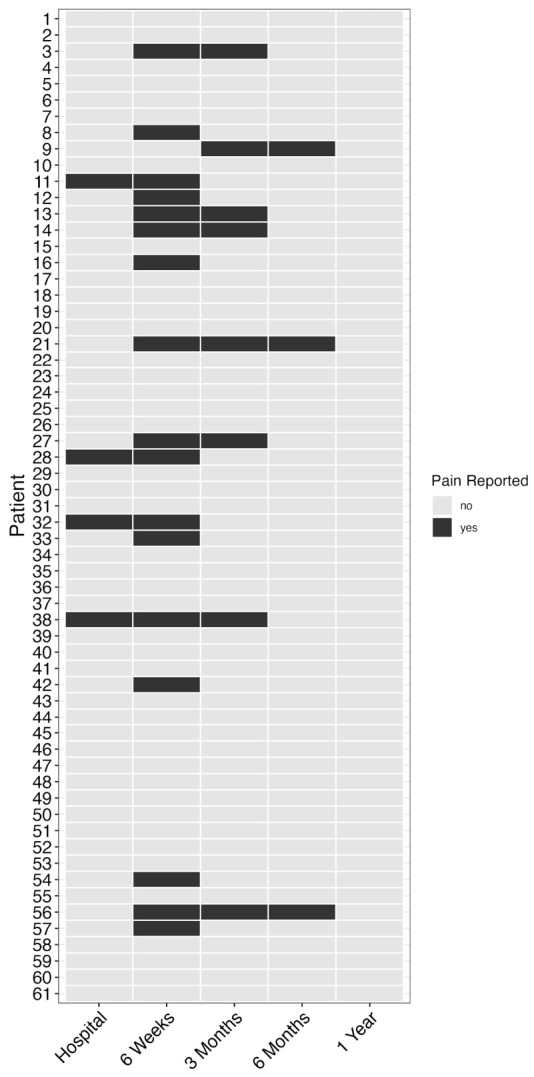
Postoperative radiculitis throughout the one-year follow-up period based on clinical records.

The overall incidence of post-operative radiculitis was 29.5% (CI 95% 18.1–41.0%) (18/61). A review of the clinical timeline for these 18 patients revealed a characteristic trajectory. While 22.2% (4/18) of cases were diagnosed in-hospital, the prevalence peaked sharply at the 6-week follow-up (94.4%; 17/18). The radiculitis was transient, with symptom prevalence decreasing to 44.4% (8/18) by 3 months and 16.6% (3/18) by 6 months. No active cases remained at the one-year follow-up (Figure 2).

The inter-observer reliability of the radiological measurements was good, with intraclass correlation coefficients (ICC) found to range from 0.87 to 0.94.

Although no significant differences were observed between Non-Pain and Pain groups regarding demographic characteristics, treated level, posterior fixation use, or anterior cage height (Table 1), the Pain group exhibited a trend toward longer hospital stay (4.3 vs. 3.1 days).

**Figure 3:**
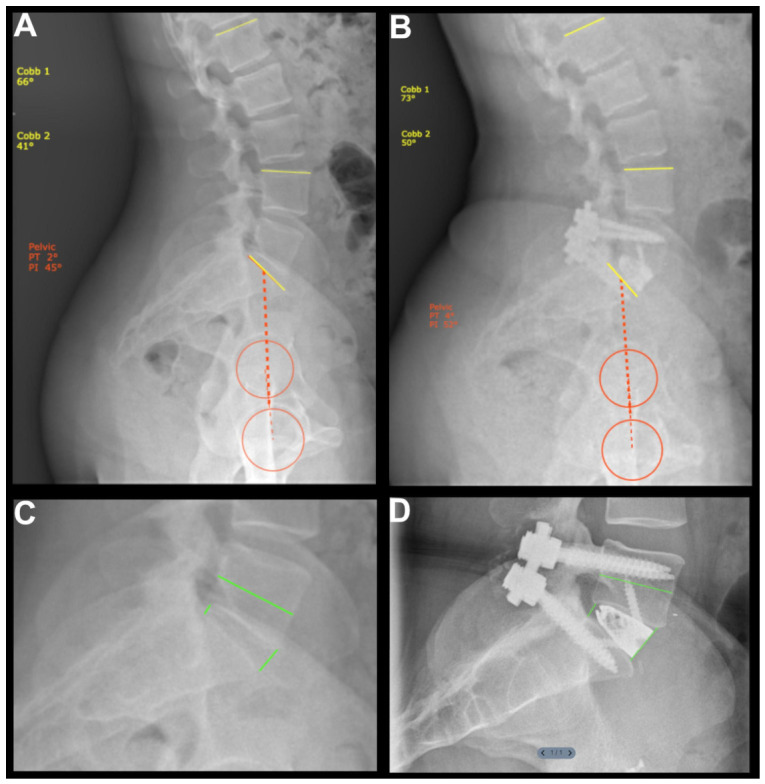
Representative lumbar sagittal radiographs of a patient in the Pain group. (**A**) Measured preoperative sagittal lumbopelvic values yielded a Relative Lumbar Lordosis (RLL) of 8°. (**B**) Postoperatively, RLL shifted to 14°. (**C**) Preoperative Anterior Disc Height (ADH) ratio was 0.32. (**D**) Postoperative ADH ratio was 0.59.

Significant differences in preoperative radiological variables were found between the Non-Pain and Pain groups for PT (16.9° vs. 11.7°, *p* = 0.03), RLL (−10.6° vs. −2.9°, *p* = 0.01), and RPV (−5.4° vs. −1.3°, *p* = 0.03) (Table 2 and Figure 3). These data indicate that, before surgery, patients in the Non-Pain group showed signs of malalignment, with elevated RLL and RPV values. Furthermore, this group’s PT was high, which suggests a mechanism of pelvic retroversion to compensate for the malalignment. In contrast, the Pain group exhibited ideal alignment parameters. Postoperatively, the groups also differed significantly in LL (54.8° vs. 63.3°, *p* = 0.02), L4S1 (37.9° vs. 45.5°, *p* < 0.001), ADH ratio (0.5 vs. 0.6, *p* = 0.003), RLL (−5.7° vs. 5.2°, *p* = 0.002), and RPV (−2.3° vs. 4.7°, *p* = 0.003) (Table 2). The Pain group had a greater LL, L4–S1 lordosis and instrumented anterior disc height than the Non-Pain group. These findings indicate that, following surgery, in the Non-Pain group, the pelvic retroversion had returned to adequate alignment, whereas a tendency towards pelvic anteversion was observed in the Pain group. Similarly, while in the Non-Pain group the preoperative relative hypolordosis (RLL −5.7°) had improved, in the Pain group the RLL had increased, showing a tendency towards hyperlordosis.

The ODI score showed no significant difference between the groups, both before surgery (Non-Pain: 33.2 vs. Pain: 32.0, *p* = 0.8) and at follow-up (Non-Pain: 10.1 vs. Pain: 14.7, *p* = 0.3).

Univariable logistic regression identified several factors associated with postoperative radiculitis (Table 3). To determine the multivariable model, only parameters demonstrating statistical significance (*p* ≤ 0.05) were considered. Additionally, the analysis of differences between pre- and postoperative values for sagittal alignment yielded no significant differences within the groups.

Postoperative ADH ratio demonstrated the highest association with the outcome (OR: 2.61, 95% CI: 1.37 to 5.66, *p* = 0.007) (Table 3). To ensure model parsimony and adhere to the “event per variable” rule, a maximum of two variables were prioritized for the final multivariable construct. The postoperative RLL was selected alongside the postoperative ADH ratio, which yielded the most robust model fit according to the AIC (64.37) and VIF (1.01) (Table 4). In this context, variables were standardized to assess the clinical impact of a one-standard-deviation increase. Postoperative RLL was confirmed as a significant, independent associated factor with the manifestation of postoperative radiculitis (OR: 2.47, 95% CI: 1.15 to 5.31, *p* = 0.02). While postoperative ADH ratio trended toward positive relationship, it did not retain independent statistical significance (OR: 1.89, 95% CI: 0.92 to 3.92, *p* = 0.083) (Table 5).

## 4. Discussion

The current study aimed to determine the incidence of postoperative radiculitis following single-segment L4–L5 or L5–S1 ALIF and to evaluate the relationship between patient sagittal alignment (assessed via GAP score components) and the likelihood of developing this complication. We observed an overall postoperative radiculitis incidence of 29.5%. Symptoms predominantly emerged around the six-week postoperative mark, gradually resolving over time, with no active cases remaining at the one-year follow-up. Analysis revealed that patients who developed radiculitis had preoperative lumbopelvic profiles, specifically RLL and RPV, that were already close to ideal alignment. Following surgery, this group was pushed toward hyperlordosis and pelvic anteversion.

A multivariable logistic regression analysis subsequently confirmed postoperative RLL as a significant independent factor, after adjusting for the ADH ratio. Although the ADH ratio itself did not reach statistical significance, its inclusion was essential to optimize the model’s performance in estimating event occurrence.

The methodology used to determine the incidence of postoperative radiculitis could be subject to debate. The application of NLP to the specific study of postoperative complications in spine surgery appears to be novel. Previous studies on this topic typically relied on manual retrospective reviews rather than automated, privacy-compliant NLP pipelines [4,17]. Structured information extraction from unstructured clinical records facilitates data accessibility for clinical research. Manual extraction by experts is time-consuming and expensive, limiting scalability. Recent clinical research increasingly validates the established methodology of using LLaMA. This shift toward using open-source LLMs is a key part of ensuring data privacy by enabling the local, on-site processing of sensitive clinical information [18]. Future research should focus on determining the method’s reliability and accuracy in detail. A recent study [19] reported that the LLaMA 3.1 model demonstrated high accuracy (94.7%) in extracting structured information from breast cancer histopathology reports, closely matching the accuracy achieved by a trained human annotator (95.4%).

Our reported incidence of 29.5% aligns closely with existing literature that utilizes similar definitions of non-structural postoperative radicular pain, such as Griffith et al. [17] (36.2%) and Araghi et al. [4] (34%). When considering studies that expand their definition to include objective structural nerve root injuries, rates exhibit much wider variance, ranging from 6.3% to 28% [6,7,20]. The rate of neurological injury in our study (3.2%) is comparable to that reported in other studies [2]. Our symptom trajectory analysis found few cases during hospitalization, with a significant increase in prevalence observed by the six-week postoperative period. This suggests an initial “silent period”, which likely contributes to underreporting [21]. This delayed symptom manifestation could result from the pain masking of multimodal analgesia [21], particularly the routine immediate postoperative use of an elastomeric pump.

The pathophysiological mechanism underlying purely sensory symptoms remains debatable. Causal mechanisms suggested in the literature include stretch neuropraxia [20], nerve root compression caused by pre-existing posteroinferior osteophytes [7], and foraminal S1 root impingement (known as the pincer mechanism) resulting from the superior articular facet after significant lordotic correction [6].

Although our analysis found no association between radiculitis onset and posterior height restoration, as assessed by the PDH ratio, we cannot definitively rule out subtle structural compression or foraminal narrowing as an explanation for the radicular pain, given that advanced imaging studies were performed only in patients with immediate postoperative pain.

While exploratory and requiring further validation, our findings highlight the utility of GAP score component analysis for the nuanced assessment of lumbopelvic alignment in short-segment lumbar fusion. These results suggest a cautionary note: striving for maximum interbody implant size, in accordance with current surgical trends, may inadvertently escalate postoperative morbidity.

Several limitations must be acknowledged when interpreting these results. First, the identification of postoperative radiculitis relied heavily on subjective patient self-reporting, and we lacked a standardized framework for monitoring pain intensity across regular clinical visits. Second, although basic radiographs and clinical exams were used to rule out major structural abnormalities, advanced cross-sectional imaging (CT or MRI) were performed only in cases presenting with immediate postoperative pain. Consequently, we could not definitively exclude minor structural nerve root pathologies in the postoperative period. Third, potential non-structural etiologies, such as chemical nerve irritation from bone grafts, especially associated with rhBMP-2 [17,22] or pre-existing nerve sensitization (e.g., preoperative steroid use), were not analyzed because none of these drugs were used at any point. Finally, the retrospective design and relatively small sample size (n = 61) inherently restrict statistical power and may introduce selection bias, limiting the overarching generalizability of the findings.

Despite the small cohort size, the findings are highly relevant to spine surgeons utilizing ALIF for lumbosacral fusion. We consider postoperative radiculitis as a cause for clinical concern. Patients experiencing this condition, which can last up to six months post-surgery and manifests as significant discomfort in our records, exhibited a trend toward longer hospital stays, though this was not statistically significant. Furthermore, our clinical impression suggests these patients expressed greater worry about surgical outcomes and required more unscheduled consultations. This is important because postoperative pain is known to significantly affect patient satisfaction and regret following surgery [23].

Surgeons should exercise caution when considering the implantation of a hyperlordotic cage in patients who already have near-ideal lumbopelvic alignment. The data indicate a potential for adverse outcome, as excessive disc space enlargement and hyperlordotic correction can be detrimental in these cases. Future studies with a larger patient cohort are needed to confirm both the validity of these GAP score thresholds and the utility of a straightforward measure of over-distraction, such as the ratio of anterior disc height to vertebral body width.

## 5. Conclusions

Postoperative radiculitis is an iatrogenic complication that may occur following ALIF, potentially related to disc space enlargement in patients who already have near-ideal lumbar alignment. For this population, caution is advised, and the degree of correction should be tailored to the individual. Utilizing parameters from the GAP score may help inform the preoperative planning.

## Figures and Tables

**Table 1:** Patient characteristics and surgical variables.

Variable	Total	Group		*p*-Value
	(n = 61)	Non-Pain (n = 43)	Pain (n = 18)	
Age	47.5 ± 9.1	48.1 ± 9.2	46.2 ± 8.9	0.459
Female sex	37 (60.7)	26 (60.5)	11 (61.1)	1.000
Current smoker	16 (26.2)	11 (25.6)	5 (27.8)	0.837
BMI	25.1 ± 4.4	25.1 ± 4.8	25.1 ± 3.5	0.972
Diabetes	2 (3.2)	2 (4.7)	0 (0.0)	1.000
Posterior fixation	40 (65.6)	27 (62.8)	13 (72.2)	0.680
Treated Level				0.084
L4–L5	13 (21.3)	12 (27.9)	1 (5.6)	
L5–S1	48 (78.7)	31 (72.1)	17 (94.4)	
Cage Ant. Height (mm)	14.9 ± 1.8	14.8 ± 1.7	15.4 ± 1.8	0.198
Hospital Stay (days)	3.4 ± 2.2	3.1 ± 1.3	4.3 ± 3.6	0.068

Values are presented as number of cases (%) or mean ± standard deviation unless otherwise indicated. BMI, Body mass index.

**Table 2:** Preoperative and Postoperative Comparison.

Phase	Variable	Group		Mean Diff.	*p*-Value
		Non-Pain (n = 43)	Pain (n = 18)		
Preoperative	ODI	33.2 ± 19.8	32.0 ± 15.1	1.2	0.812
	PI (°)	50.8 ± 13.1	47.1 ± 11.1	3.7	0.268
	PT (°)	16.9 ± 10.7	11.7 ± 7.6	5.2	**0.036**
	SS (°)	33.6 ± 9.0	35.4 ± 9.0	−1.8	0.464
	LL (°)	49.7 ± 12.9	55.3 ± 12.8	−5.6	0.135
	L4S1(°)	29.1 ± 9.2	34.3 ± 7.2	−5.2	0.054
	ADH ratio	0.2 ± 0.1	0.2 ± 0.1	0.0	0.928
	PDH ratio	0.1 ± 0.0	0.1 ± 0.1	0.0	0.470
	RLL (°)	−10.7 ± 12.4	−2.9 ± 10.3	−7.8	**0.015**
	RPV (°)	−5.4 ± 7.7	−1.3 ± 6.1	−4.1	**0.034**
	LDI (%)	59.4 ± 15.3	63.6 ± 11.9	−4.2	0.248
Postoperative	ODI	10.1 ± 11.1	14.7 ± 14.3	−4.6	0.300
	PI (°)	51.6 ± 12.7	52.1 ± 9.7	−0.5	0.867
	PT (°)	15.0 ± 10.3	10.6 ± 7.5	4.4	0.066
	SS (°)	36.7 ± 9.2	41.5 ± 9.1	−4.8	0.161
	LL (°)	54.8 ± 11.8	63.3 ± 13.7	−8.5	**0.028**
	L4S1 (°)	37.9 ± 7.4	45.5 ± 6.9	−7.6	**<0.001**
	ADH Ratio	0.5 ± 0.1	0.6 ± 0.1	−0.1	**0.003**
	PDH Ratio	0.2 ± 0.0	0.3 ± 0.1	−0.1	0.337
	RLL (°)	−5.7 ± 10.8	5.2 ± 11.9	−10.9	**0.002**
	RPV (°)	−2.3 ± 8.5	4.7 ± 7.5	−7.0	**0.003**
	LDI (%)	72.0 ± 21.7	73.4 ± 10.1	−1.4	0.733

Values are presented as mean ± standard deviation. **ODI:** Oswestry Disability Index; **PI:** Pelvic Incidence; **PT:** Pelvic Tilt; **SS:** Sacral Slope; **LL:** Lumbar Lordosis; **L4S1:** L4–S1 Lordosis; **ADH:** Anterior Disc Height; **PDH:** Posterior Disc Height; **RLL:** Relative Lumbar Lordosis; **RPV:** Relative Pelvic Version; **LDI:** Lordosis Distribution Index.

**Table 3:** Univariable logistic regression analysis.

Variable	Estimate	OR	95% CI	*p*-Value
Postop L4S1	0.14	1.15	1.05 to 1.25	**0.003**
Postop RLL	0.09	1.10	1.04 to 1.17	**0.002**
Postop RPV	0.12	1.13	1.04 1.23	**0.006**
Postop ADH ratio	0.96	2.61	1.37 to 5.66	**0.007**
Postop LL	0.06	1.06	1.01 to 1.12	**0.021**
Preop RLL	0.06	1.06	1.01 to 1.12	**0.028**
Preop L4S1	0.07	1.07	1.00 to 1.15	**0.044**

**Table 4:** AIC for multivariable logistic regression models adding each variable to a model already containing Postop ADH ratio.

Added Variable	AIC
Postop RLL	64.37
Postop L4S1	65.93
Postop RPV	66.45
Postop LL	68.99
Preop RLL	69.5
Preop L4S1	70.27

**Table 5:** Final multivariable logistic regression model.

Variable	Estimate	OR	95% CI	*p*-Value
Postop RLL	0.91	2.47	1.15 to 5.31	**0.020**
Postop ADH ratio	0.64	1.89	0.92 to 3.92	0.082

## Data Availability

The data presented in this study are available on request from the corresponding author.

## Supplementary Materials

The following supporting information can be downloaded at: https://www.mdpi.com/article/10.3390/jcm15124707/s1, Supplemental Materials S1: Local Large Language Model (LLM) Methodology.

## Abbreviations

The following abbreviations are used in this manuscript:

ADH	Anterior Disc Height
AIC	Akaike Information Criterion
ALIF	Anterior Lumbar Interbody Fusion
AP	Anteroposterior
BMI	Body Mass Index
CT	Computed Tomography
GAP	Global Alignment and Proportion
ICC	Intraclass Correlation Coefficients
L4S1	L4-S1 Lordosis
LDI	Lordosis Distribution Index
LL	Lumbar Lordosis
LLM	Large Language Model
MRI	Magnetic Resonance Imaging
NLP	Natural Language Processing
ODI	Oswestry Disability Index
PDH	Posterior Disc Height
PI	Pelvic Incidence
PT	Pelvic Tilt
RLL	Relative Lumbar Lordosis
RPV	Relative Pelvic Version
SS	Sacral Slope
VIF	Variance Inflation Factor

## References

[1] Mobbs R.J., Loganathan A., Yeung V., Rao P.J. (2013). Indications for Anterior Lumbar Interbody Fusion. Orthop. Surg..

[2] Fujii T., Kumar R., Cha J., Bansal A., De Oliveira R.G., Louie P.K., Nemani V.M., Leveque J.-C., Sethi R.K. (2025). Neurological Complications Following Anterior Lumbar Interbody Fusion (ALIF): A Systematic Review. Glob. Spine J..

[3] Boakye L.A.T., Fourman M.S., Spina N.T., Laudermilch D., Lee J.Y. (2018). “Post-Decompressive Neuropathy”: New-Onset Post-Laminectomy Lower Extremity Neuropathic Pain Different from the Preoperative Complaint. Asian Spine J..

[4] Araghi K., Fourman M.S., Merrill R.K., Maayan O., Zhao E., Pajak A., Subramanian T., Kim D.N., Kamil R., Shahi P. (2023). Postoperative Radiculitis After L5-S1 Anterior Lumbar Interbody Fusion. Spine.

[5] Goldman S.N., Xavier J., Rothchild E., Song H., Hui A.T., Parker-Fong K., Schwartz R., Schlumprecht A., Akioyamen N.O., Gelfand Y. (2025). Establishing a Common Term and Definition for Non-Structural Postoperative Radicular Pain Following Lumbar Decompression: A Scoping Review. HSS J. Musculoskelet. J. Hosp. Spec. Surg..

[6] Compagnone D., Langella F., Cecchinato R., Damilano M., Messina C., Sconfienza L.M., Lamartina C., Berjano P. (2022). Post-Operative L5 Radiculopathy after L5-S1 Hyperlordotic Anterior Lumbar Interbody Fusion (HL-ALIF) Is Related to a Greater Increase of Lordosis and Smaller Post-Operative Posterior Disc Height: Results from a Cohort Study. Eur. Spine J..

[7] Dowlati E., Alexander H., Voyadzis J.-M. (2020). Vulnerability of the L5 Nerve Root during Anterior Lumbar Interbody Fusion at L5–S1: Case Series and Review of the Literature. Neurosurg. Focus.

[8] Formica M., Quarto E., Zanirato A., Mosconi L., Lontaro-Baracchini M., Alessio-Mazzola M., Felli L. (2021). ALIF in the Correction of Spinal Sagittal Misalignment. A Systematic Review of Literature. Eur. Spine J..

[9] Janjua M.B., Ozturk A.K., Ackshota N., McShane B.J., Saifi C., Welch W.C., Arlet V. (2020). Surgical Treatment of Flat Back Syndrome with Anterior Hyperlordotic Cages. Oper. Surg..

[10] Nguyen A.Q., Ukogu C., Harvey J.P., Federico V.P., Nolte M.T., Khanna K., Sheha E.D., Gandhi S.D., Phillips F.M. (2023). Increased Cage Angle Effects on Radiographic Outcomes after Stand-Alone Anterior Lumbar Interbody Fusion. J. Neurosurg. Spine.

[11] Yilgor C., Sogunmez N., Boissiere L., Yavuz Y., Obeid I., Kleinstück F., Pérez-Grueso F.J.S., Acaroglu E., Haddad S., Mannion A.F. (2017). Global Alignment and Proportion (GAP) Score: Development and Validation of a New Method of Analyzing Spinopelvic Alignment to Predict Mechanical Complications After Adult Spinal Deformity Surgery. J. Bone Jt. Surg. Am..

[12] Zheng G., Wang C., Wang T., Hu W., Ji Q., Hu F., Li J., Chaudhary S.K., Song K., Song D. (2020). Relationship between Postoperative Lordosis Distribution Index and Adjacent Segment Disease Following L4-S1 Posterior Lumbar Interbody Fusion. J. Orthop. Surg. Res..

[13] Legaye J., Duval-Beaupere G., Marty C., Hecquet J. (1998). Pelvic Incidence: A Fundamental Pelvic Parameter for Three-Dimensional Regulation of Spinal Sagittal Curves. Eur. Spine J..

[14] Protopsaltis T.S., Soroceanu A., Tishelman J.C., Buckland A.J., Mundis G.M., Smith J.S., Daniels A., Lenke L.G., Kim H.J., Klineberg E.O. (2020). Should Sagittal Spinal Alignment Targets for Adult Spinal Deformity Correction Depend on Pelvic Incidence and Age?. Spine.

[15] Heinze G., Schemper M. (2002). A Solution to the Problem of Separation in Logistic Regression. Stat. Med..

[16] Zabor E.C., Reddy C.A., Tendulkar R.D., Patil S. (2022). Logistic Regression in Clinical Studies. Int. J. Radiat. Oncol. Biol. Phys..

[17] Griffith M.S., Shaw K.A., Burke B.K., Jackson K.L., Gloystein D.M. (2021). Post-Operative Radiculitis Following One or Two Level Anterior Lumbar Surgery with or without Posterior Instrumentation. J. Orthop..

[18] Wals Zurita A.J., Miras Del Rio H., Ugarte Ruiz De Aguirre N., Nebrera Navarro C., Rubio Jimenez M., Muñoz Carmona D., Miguez Sanchez C. (2025). The Transformative Potential of Large Language Models in Mining Electronic Health Records Data: Content Analysis. JMIR Med. Inform..

[19] Balasubramanian J.B., Adams D., Roxanis I., De Gonzalez A.B., Coulson P., Almeida J.S., García-Closas M. (2025). Leveraging Large Language Models for Structured Information Extraction from Pathology Reports. J. Pathol. Inform..

[20] Singh M., Schmitt P., Melvani N., Nassar J., Farias M., Diebo B., Daniels A. (2025). Stretch Neuropraxia after L5-S1 Anterior Lumbar Interbody Fusion: Effect of Cage Height and Lordosis. Eur. Spine J..

[21] Meyrat R., Vivian E., Sridhar A., Gulden R.H., Bruce S., Martinez A., Montgomery L., Reed D.N., Rappa P.J., Makanbhai H. (2023). Development of Multidisciplinary, Evidenced-Based Protocol Recommendations and Implementation Strategies for Anterior Lumbar Interbody Fusion Surgery Following a Literature Review. Medicine.

[22] Lee D.D., Kim J.Y. (2017). A Comparison of Radiographic and Clinical Outcomes of Anterior Lumbar Interbody Fusion Performed with Either a Cellular Bone Allograft Containing Multipotent Adult Progenitor Cells or Recombinant Human Bone Morphogenetic Protein-2. J. Orthop. Surg. Res..

[23] Berkowitz R., Vu J., Brummett C., Waljee J., Englesbe M., Howard R. (2021). The Impact of Complications and Pain on Patient Satisfaction. Ann. Surg..

