# Supplementary material for: Sagittal Alignment Correction and the Extent of Intervertebral Distraction as Factors Associated with Postoperative Radiculitis Following Anterior Lumbar Interbody Fusion
Source: J Clin Med. 2026 Jun 17;15(12):4707. doi: 10.3390/jcm15124707 (PMC13302206; doi:10.3390/jcm15124707)
Supplement: Supplementary file 1 [file jcm-15-04707-s001.zip › jcm-4282052-supplementary.pdf]

Sagittal Alignment Correction and the Extent of Intervertebral Distraction as Factors Associated with Postoperative Radiculitis Following Anterior Lumbar Interbody Fusion.

Paula Lavezzolo1, Francesco Caiazzo1, Lucas Capo1, Andreas Leidinger1, Daniel Alveal-Mellado2, Judith Salat-Batlle2, and Juan Bago1

1 Instituto Quirúrgico Spanò, Sagrada Familia Medical Center, Barcelona, Spain
2 Fundación Privada Spanò, Barcelona, Spain
Correspondence: jbago@iqspano.com (J. Bago)

Supplemental materials S1: Local Large Language Model (LLM) Methodology

Contents: LLM Configuration, Prompt Architecture, Golden Examples, and Python Execution Script.

1. AI Model and Environment Configuration

- **Local Engine:** Ollama (v0.23.0)
- **Base Model:** llama3.1 8B
- **Hyperparameters:** Temperature = 0
- **Environment:** Python 3.14.4

2. Prompt Architecture

The Local Large Language Model (LLM) was tasked with acting as a senior medical auditor. The model was instructed to analyze longitudinal, chronologically ordered clinical histories and extract specific pre-operative, intra-operative, and post-operative variables. The exact few-shot prompt template used in the script is detailed below.

2.1. System and Task Instructions (Original Spanish Prompt)

> Actúa como Auditor Médico Senior con extenso conocimiento en lenguaje y abreviaciones médicas. Especialista en cirugía de columna lumbar. Analiza el historial completo del paciente (ordenado por fecha, que se encuentra en la columna Clinical_Note_Text).
>
> **TAREA DE EXTRACCIÓN:**
> 1. Localiza el segmento operado (L5-S1 o L4-L5). Si no es claro, deja la celda en blanco.
> 2. Analiza datos PREVIOS (Pre-Op) y POSTERIORES (Post-Op). Estima si la visita es pre o postoperatoria basándose en el texto de la misma.

2.2. Extraction Variables and JSON Schema

EXTRAE ESTAS VARIABLES EXACTAS (Formato JSON):

--- BLOQUE 1: PERFIL PRE-OPERATORIO ---

1. "Pain_Duration_PreOp": ¿Cuánto tiempo llevaba con dolor antes de operarse? (Ej: "6 meses", "2 años", "Larga data"). Si no dice, "Not stated".
2. "Pain_Type_PreOp": ¿Qué predominaba? (Lumbar/Piernas/"Not Stated").
3. "Previous_Spine_Surgery": ¿Había sido operado de columna lumbar ANTES de este episodio? (Yes/No).
4. "Motor_Weakness_Pre": ¿Había compromiso motor antes de la cirugía? (Yes/No)
5. "Diagnostico_Clinico": Cuál es el diagnóstico clínico principal (Enfermedad Discal Degenerativa, Espondilolistesis, Estenosis Foraminal, Estenosis central, Otra (especificar))

--- BLOQUE 2: MEDICACIÓN (Analiza si tomaba alguna medicación ANTES de la cirugía) ---

6. "Medicacion": ¿Tomaba analgésicos simples/opioides? (Paracetamol, Tramadol, Nolotil...); ¿Tomaba AINEs [Antiinflamatorios No Esteroideos]? (Enantyum, Ibuprofeno, Diclofenaco...); ¿Tomaba Corticoides/Infiltraciones? (Celestone, Inzitan, Dexametasona...)

--- BLOQUE 3: RESULTADO POST-OPERATORIO ---

7. "PostOp_Complication": ¿Hubo alguna complicación? (Nueva cirugía del mismo segmento, Migración de material, desgarro dural, hematoma, ninguno).

8. "PostOp_Satisfaction": ¿El paciente expresa estar mejor/contento al final o luego de la cirugía? (Happy / Not Happy / Unclear).

9. "Radiculitis": ¿El paciente manifiesta dolor postoperatorio en alguna o ambas piernas? ¿Este dolor lo describe como quemante, de tracción o en el recorrido de alguna raíz lumbar L4-S1? Busca también palabras como neuritis o radiculitis. EJEMPLO: "...ha tenido neuritis por estiramiento radicular"; "...compatible con neuritis en ambas piernas"; "dolor en las piernas"

10. "Motor_Weakness_Post": ¿El paciente manifiesta pérdida de fuerza o compromiso motor tras la cirugía?

Responde SOLO con este JSON: { "Pain_Duration_PreOp": "...", "Pain_Type_PreOp": "...", "Previous_Spine_Surgery": "...", "Motor_Weakness_Pre":"...", "Diagnostico_Clinico":"...", "Medicacion": "...",

"PostOp_Complication": "...", "PostOp_Satisfaction": "...", "Radiculitis":"...", "Motor_Weakness_Post":"..." }

3. Golden Examples (Few-Shot Prompting)

### EJEMPLO DE HISTORIAL CLÍNICO ### [2021-01-15]: Paciente acude por dolor lumbar irradiado a pierna derecha de 8 meses de evolución. Tomando Tramadol y Enantyum. No cirugías previas de columna. Fuerza conservada. Diagnóstico: Hernia discal L4-L5. [2021-02-10]: Intervenido hace 3 semanas de ALIF L4-L5. Refiere mejoría notable del dolor radicular, muy contento con el resultado. Sin complicaciones (no desgarro dural). No pérdida de fuerza en MMII. ### RESPUESTA ESPERADA PARA EL EJEMPLO (JSON) ###
{ "Pain_Duration_PreOp": "8 meses", "Pain_Type_PreOp": "Lumbar y Piernas", "Previous_Spine_Surgery": "No", "Motor_Weakness_Pre": "No", "Diagnostico_Clinico": "Hernia discal", "Medicacion": "Tramadol, Enantyum", "PostOp_Complication": "Ninguno", "PostOp_Satisfaction": "Happy", "Radiculitis": "No", "Motor_Weakness_Post": "No" } """
